# Synthetic Spectrogram Augmentation via Semi-Supervised WGAN-GP for Acoustic Industrial Quality Inspection of Turbine Housings Under Extreme Data Scarcity

**DOI:** 10.3390/s26103052

**Published:** 2026-05-12

**Authors:** Ander Gracia Moisés, Óscar Del Barrio Farran, David Martinez García, María Puy Zudaire Latienda

**Affiliations:** NAITEC—Fundación I + D Automoción y Mecatrónica, Calle Tajonar 20, 31006 Pamplona, Spain

**Keywords:** acoustic NDT, audio spectrograms, data augmentation, semi-supervised learning, generative adversarial networks, industrial quality inspection, turbine housings

## Abstract

Impact-based acoustic inspection provides a rapid non-destructive approach for screening metallic components by analyzing the sound radiated after a controlled mechanical excitation. However, the limited availability of labeled data from defective parts remains a major challenge for deploying deep learning classifiers in production. This paper proposes a complete pipeline that converts raw impact-response audio recordings into magnitude log-spectrogram images and trains a semi-supervised Wasserstein GAN with gradient penalty (SS-WGAN-GP) designed to operate under extreme data scarcity. The architecture couples a shared convolutional backbone with two output heads: a Wasserstein critic for unsupervised discrimination between real and generated samples, and a binary classification head for supervised quality labeling, jointly optimized through a combined loss that balances Wasserstein distance, gradient penalty, and cross-entropy. A key property of the design is that the generator acts as a source of synthetic training samples, producing progressively more realistic spectrograms as training advances. These samples, in turn, enrich the feature representations learned by the shared backbone and improve the performance of the classification head. The classification head of the trained discriminator is deployed directly as the quality classifier, without requiring external data or post hoc retraining.

## 1. Introduction

Acoustic non-destructive testing (NDT) is a well-established approach for structural damage detection in metallic components through the analysis of the acoustic response generated by controlled mechanical excitation [1,2]. The dynamic properties of a structure—natural frequencies, mode shapes, and damping coefficients—are governed directly by its physical parameters, principally stiffness, mass, and internal continuity. Defects such as cracks or porosity reduce local stiffness, thereby modifying the resonant behavior of the structure and, consequently, its radiated acoustic response in a repeatable and measurable manner, without contact, consumables, or disassembly [3]. In the context of turbocharger remanufacturing, where used components are inspected and selectively restored to give them a second life, this property makes acoustic impact testing an attractive early quality-inspection stage: conforming and defective turbine housings (THs) subjected to the same impact conditions produce distinct acoustic signatures that can be detected at the beginning of the remanufacturing workflow. The TH samples used in this study were provided by BORG Automotive Reman, a company with a circular business model based on remanufacturing high-quality auto spare parts.

Early identification of non-conforming parts has important economic and environmental implications. If a defect is detected only after a component has already passed through several downstream remanufacturing stages, the value added by those operations is lost and additional costs arise from reprocessing, material waste, and production downtime. Because destructive inspection cannot be applied to every part and many conventional inspection methods remain too slow or too costly for in-line deployment, rapid non-contact acoustic inspection techniques with minimal sample preparation are especially attractive in this setting [4].

Despite these advantages, the adoption of machine learning for acoustic inspection remains constrained by the scarcity of labeled defective-part data. Reliable annotation requires controlled acquisition and expert validation, while defective samples are inherently less available than conforming ones. Under these conditions, the amount of data available is often insufficient to train deep learning models robustly, which motivates the use of methods capable of learning effectively under limited supervision.

Generative Adversarial Networks (GANs) [5] have emerged as a promising approach for data augmentation in low-data regimes, with applications in medical imaging [6], remote sensing [7], spectroscopic data [8], and acoustic signal processing [9]. The Wasserstein GAN with gradient penalty (WGAN-GP) [10,11] addresses the training instability and mode collapse typical of GANs, while semi-supervised GANs (SGANs) [12] extend the adversarial framework by incorporating class labels into the discriminator. This enables simultaneous discrimination between real and generated samples and supervised feature learning, which has been shown to improve both generation quality and downstream classification performance in low-data scenarios [13].

In this work, we present a framework for binary quality inspection of THs that combines the training stability of WGAN-GP with the label-efficient representation learning of semi-supervised GANs in a unified semi-supervised WGAN-GP (SS-WGAN-GP) architecture. The proposed approach transforms raw impact-response recordings into magnitude log-spectrogram images and learns a shared representation that supports both adversarial generation and supervised defect classification.

The main contributions of this work are threefold. First, a semi-supervised WGAN-GP architecture is proposed for acoustic quality inspection under extreme data scarcity, combining a Wasserstein critic and a binary classification head within a shared discriminator. Second, the trained discriminator is used directly as the final quality classifier, avoiding the need for auxiliary classifiers or post hoc retraining. Third, the interaction between adversarial learning and supervised classification through the shared backbone is analyzed to explain why the proposed framework can remain trainable even when only a very small number of labeled samples is available. The remainder of this paper is organized as follows. Section 2 reviews the related work. Section 3 presents the methodology, including audio acquisition, spectrogram generation, the SS-WGAN-GP architecture, and the interaction between the adversarial and supervised branches. Section 4 reports the experimental setup and results. Section 5 and Section 6 present the discussion and conclusions, respectively.

## 2. Related Work

This section reviews the literature most relevant to the proposed approach. It covers prior work on spectrogram-based acoustic NDT, GAN-based data augmentation in industrial settings, and semi-supervised adversarial learning.

### 2.1. Acoustic NDT and Spectrogram-Based Classification

Impact-based acoustic testing analyzes the acoustic response of metallic components to short mechanical impulses and has been applied to the detection of internal defects in castings and structural elements [14]. Classical approaches rely on hand-crafted frequency-domain features such as resonant peak positions, power spectral density profiles, and quality factors [15]. More recent work has shifted toward learned representations, using spectrograms—time–frequency images computed via the Short-Time Fourier Transform—as input to convolutional neural networks, thereby exploiting the rich spatial structure of spectral content and temporal decay [16]. This choice of representation is particularly natural for impact responses, where defect signatures appear as systematic changes in spectral content and temporal decay patterns that are visible in the spectrogram structure.

### 2.2. GANs for Industrial Data Augmentation

GAN-based data augmentation has been applied to surface defect detection [17], bearing fault diagnosis [18], and acoustic emission monitoring [19], among other industrial applications [20]. However, much of the existing literature assumes settings in which a moderate number of labeled samples is available, whereas scenarios with extremely scarce labeled data have received comparatively less attention.

In this context, WGAN [21] and its gradient-penalty variant WGAN-GP [22] are particularly relevant because they provide more stable training dynamics than the original GAN formulation. WGAN replaces the Jensen–Shannon divergence with the Wasserstein-1 distance, which yields more informative gradients when the generated and real data distributions have little overlap. WGAN-GP further improves this framework by enforcing the Lipschitz constraint through a differentiable gradient penalty, leading to more stable optimization in practice.

### 2.3. Semi-Supervised Adversarial Learning

SGANs have shown that augmenting the discriminator with a classification head enables the model to exploit large pools of unlabeled data while preserving supervised discriminative performance [23]. The present work departs from that formulation by replacing the external pool of unlabeled data with generator-produced synthetic spectrograms, which serve as the unlabeled stream during training. This design is particularly appropriate for settings in which even unlabeled target-domain samples are limited, and it underpins the mutual reinforcement mechanism described in Section 3.3.

## 3. Methodology

This section describes the methodology from raw audio acquisition to final quality classification. It first outlines the acquisition and preprocessing steps used to obtain the real spectrogram dataset. It then presents the SS-WGAN-GP architecture, including the generator, the shared backbone, the dual-head discriminator, and the training objective. Finally, it explains how adversarial learning and supervised classification interact through the shared representation learned by the model.

**Figure 1:**
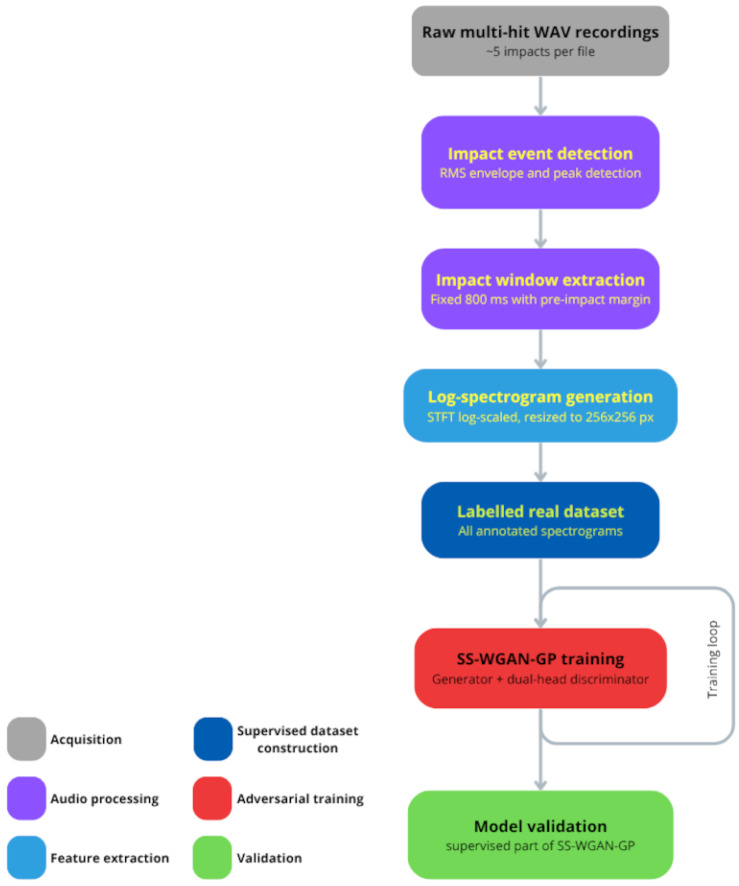
Overview of the proposed pipeline. Raw multi-hit audio recordings are segmented into individual impact events, converted to magnitude log-spectrogram images, and used to train the SS-WGAN-GP. The classification head of the trained discriminator is used directly as the quality classifier at inference time.

Figure 1 illustrates the complete pipeline. Raw multi-hit audio recordings are first segmented into individual impact events, each extracted within a fixed-length window and transformed into a magnitude log-spectrogram. The resulting labelled dataset is then used to train the SS-WGAN-GP, whose classification head serves directly as the binary quality classifier at inference time, without additional post-training steps.

### 3.1. Audio Acquisition

Acoustic data are acquired in a semi-anechoic chamber compliant with UNE-EN ISO 3745 by applying controlled hammer impacts to a TH and recording the resulting acoustic response. Two quality categories are considered: conforming THs and defective THs with known structural defects. The defective THs used in this study are remanufactured turbocharger components that were rejected by the industrial partner (BORG Automotive Reman) during their quality-control process. Because these parts have already undergone intensive service, the defects are associated with operational wear and include cracks, critical surface wear, and material loss resulting from breakage. The parts arrive pre-labeled by the industrial partner according to their accept/reject criteria; the present study does not independently characterize the defect type, location, or severity. The model is therefore a binary screening tool validated for the defect population represented in this dataset, not a defect-characterization or defect-sizing system.

**Figure 2:**
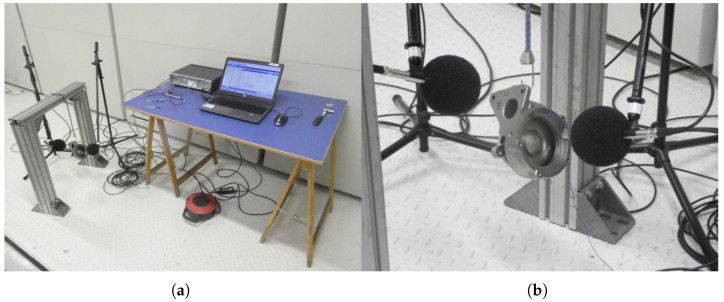
Experimental setup for acoustic data acquisition inside a semi-anechoic chamber. (**a**) Overall view of the acquisition system, showing the instrumented frame holding the TH, the two PCB 378B02 microphones mounted on tripods, and the Simcenter SCADAS Mobile front-end connected to a laptop running Simcenter Testlab software. (**b**) Close-up view of the TH suspended between the two microphones, illustrating the relative position of the microphones with respect to the component.

The acquisition system consists of two PCB 378B02 microphones (PCB Piezotronics, Inc., Depew, NY, USA) and a Simcenter SCADAS Mobile (Siemens Digital Industries Software, Plano, TX, USA) front-end controlled through Simcenter Testlab software (version 2019.1). Excitation is applied with an instrumented PCB 086E80 high-frequency impact hammer (PCB Piezotronics, Inc., Depew, NY, USA), which covers the frequency range of interest for the structural response of the TH. The impact hammer produces a short-duration broadband mechanical excitation that induces free vibration of the turbine housing. The resulting acoustic response is characterized by a high-energy onset followed by a decaying ring-down response, whose spectral content and decay rate depend on the structural properties of the component. Each detected impact event was extracted using a fixed-length window of 800 ms with a pre-impact margin of 30 ms, allowing the initial transient and subsequent decay to be captured consistently across samples. The acquisition was performed at a sampling frequency of 40,960 Hz. The usable frequency range of the PCB 086E80 impact hammer (PCB Piezotronics, Inc., Depew, NY, USA) extends up to 100 kHz, while the effective measurement bandwidth in this test was limited by the acquisition bandwidth and microphone response. The PCB 378B02 microphones cover the range from 10 Hz to 15 kHz, and were positioned at approximately 25 cm from the TH. The complete acquisition setup is shown in Figure 2.

Although the acquired signal is acoustic, the discriminative information originates in the structural dynamics excited by the impact. As a simplified illustration, the natural frequency of a single-degree-of-freedom system is given in Equation (1). This expression is not a mechanical model of the THs, which is a complex structure with numerous coupled vibration modes. It is included only to motivate the general physical principle: defects such as cracks or material loss reduce local stiffness, alter damping and modal coupling, and consequently shift the resonant frequencies and modify the radiated acoustic response with respect to the intact state. The proposed classification approach does not rely on this simplified representation. Instead, the convolutional backbone learns discriminative time–frequency patterns directly from the measured spectrograms, without assuming any analytical model of the structural dynamics. The resulting changes in the acoustic response are therefore exploited in a data-driven manner to discriminate conforming from defective THs before any downstream remanufacturing operation.

(1) $$f=\frac{1}{2\pi }\sqrt{\frac{k}{m}}$$

where *f* is the natural frequency, *k* is the equivalent stiffness, and *m* is the effective mass. The presence of a crack reduces local stiffness and therefore lowers the effective value of *k*. Since the mass of the component remains approximately constant, this stiffness reduction shifts the resonant frequencies of the structure with respect to its intact state. Defects can also alter the modal behavior of the component and, consequently, its radiated acoustic response. These changes are reflected in the recorded impact-response audio and can therefore be used to discriminate conforming from defective THs before any downstream remanufacturing operation.

**Figure 3:**
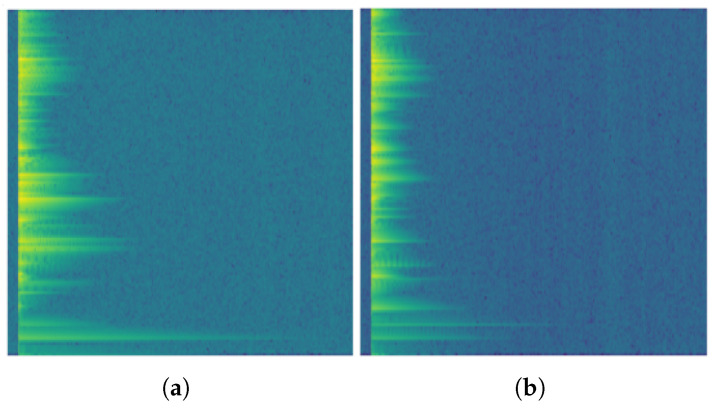
Representative 256×256 magnitude log-spectrogram images of a TH rendered with the viridis colormap: (**a**) conforming sample and (**b**) defective sample. The horizontal axis represents time after impact, the vertical axis represents frequency, and the color scale represents log-magnitude spectral energy, with darker colors indicating lower energy and yellowish colors indicating higher energy.

Each segmented impact-response event is transformed into a two-dimensional time–frequency representation using the Short-Time Fourier Transform. The magnitude spectrogram is then log-scaled to compress its dynamic range, as illustrated in Figure 3. A fixed-size 256×256 image representation is adopted to preserve the main spectral structure and temporal decay patterns while keeping the model input manageable, which is beneficial for training under limited-data conditions. This procedure yields the dataset summarized in Table 1.

### 3.2. SS-WGAN-GP Architecture

**Figure 4:**
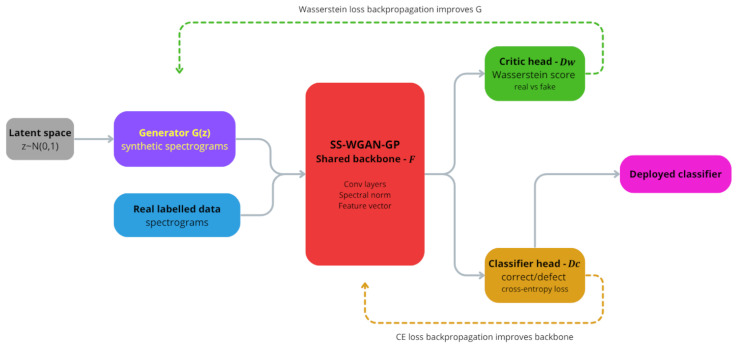
SS-WGAN-GP training architecture for spectrogram generation and classification. The colors identify the main functional blocks: latent input, generator, real labelled data, shared backbone, critic head, classifier head, and deployed classifier; dashed arrows indicate the adversarial and classification loss backpropagation paths.

The proposed architecture consists of three networks: a generator *G*, a shared convolutional backbone *F*, and two output heads attached to *F*: a Wasserstein critic head $D_{w}$ and a binary classification head $D_{c}$, as shown in Figure 4.

#### 3.2.1. Generator

The generator $G:R^{100}\to R^{1\times 256\times 256}$ maps a 100-dimensional latent vector z∼N(0,I) to a single-channel spectrogram image through a sequence of seven transposed-convolutional stages that progressively upsample from a 4×4 seed, with channel counts 512→256→128→64→32→16→1, each followed by Batch Normalization and ReLU activation except for the final layer, which applies tanh to constrain the outputs to [−1,1]. All convolutional and normalization layers are initialized with a Gaussian distribution N(0,0.02).

#### 3.2.2. Shared Backbone and Dual-Head Discriminator

The shared backbone *F* processes input images—either real labeled spectrograms or generator outputs—through six convolutional layers with 64, 128, 256, 512, 1024, and 2048 channels, each applying Spectral Normalization [24], LeakyReLU activations (α=0.2), and spatial downsampling by a factor of two. Dropout with probability 0.25 is applied after every layer except the first to regularize feature extraction given the small training set. The resulting 2048×4×4 tensor is flattened and linearly projected to a 256-dimensional feature vector. Spectral normalization is used in place of Batch Normalization because the gradient-penalty computation requires per-sample gradients, which are distorted by the batch-dependent statistics introduced by Batch Normalization.

The Wasserstein critic head $D_{w}$ is a two-layer MLP (256→128→1) that produces a single unbounded scalar, with spectral normalization applied to its hidden layer. The classification head $D_{c}$ is a two-layer MLP (256→128→2) with LeakyReLU activation and dropout (p=0.3) on the hidden layer, producing two logits corresponding to the conforming and defective classes; class probabilities are obtained via softmax and the prediction is given by the argmax. At inference time, a new spectrogram is passed through *F* and $D_{c}$, and the class with the highest softmax probability is taken as the predicted label. No retraining, fine-tuning, or additional model is required after SS-WGAN-GP training is complete.

#### 3.2.3. Training Objective

The total discriminator loss combines three terms:

(2) $$L_{D}=\underset{\mathrm{Wasserstein}\;\mathrm{loss}}{\underset{︸}{E_{z}\left[D_{w}\left(G\left(z\right)\right)\right]-E_{x}\left[D_{w}\left(x\right)\right]}}+\underset{\mathrm{Gradient}\;\mathrm{penalty}}{\underset{︸}{\lambda_{\mathrm{gp}}\;E_{\hat{x}}\;\left[(∥\nabla_{\hat{x}}D_{w}\left(\hat{x}\right){{∥}_{2}-1)}^{2}\right]}}+\underset{\mathrm{Supervised}\;\mathrm{BCE}}{\underset{︸}{\alpha_{\sup}\;\mathrm{BCE}\left(D_{c}\left(x\right),y\right)}}$$

where $\hat{x}=\epsilon x+\left(1-\epsilon \right)G\left(z\right)$ with ϵ∼U(0,1), $\lambda_{\mathrm{gp}}=10$, and $\alpha_{\sup}=1$ weights the supervised binary cross-entropy (BCE) objective. The generator loss is $L_{G}=-E_{z}\left[D_{w}\left(G\left(z\right)\right)\right]$ [25]. The critic is updated $n_{\mathrm{critic}}=5$ times per generator step, and both networks are optimized with Adam ($\beta_{1}=0.5$, $\beta_{2}=0.999$) at a learning rate of $2\times 10^{-5}$. The gradient-penalty coefficient $\lambda_{\mathrm{gp}}=10$ follows the value originally proposed in [11], while the supervised weight $\alpha_{\sup}=1$ was selected empirically as the value that yielded the best trade-off between generation quality and classification accuracy on the labeled training data.

All real spectrograms serve simultaneously as real samples for the Wasserstein loss and as labeled samples for the supervised BCE term. The generator provides the unsupervised stream: synthetic samples from G(z) feed the Wasserstein and gradient-penalty terms exclusively, while $D_{c}$ is updated only on real labeled images. This design maximizes the use of every available labeled example, which is critical when only 116 labeled samples are available.

The use of WGAN-GP is particularly important in this low-data setting because it mitigates two common failure modes of traditional GANs: vanishing gradients and mode collapse. Unlike the original GAN loss based on Jensen–Shannon divergence, the Wasserstein objective provides smoother and more informative gradients even when the real and generated distributions are initially far apart—a situation that naturally arises when the training set is extremely small. The gradient penalty further regularizes the critic by enforcing the Lipschitz constraint through a differentiable penalty term, eliminating the need for weight clipping and reducing unstable critic behavior.

### 3.3. Generator-Classifier Mutual Reinforcement

A distinctive property of SS-WGAN-GP training is the indirect interaction between the generator and the classification head through the shared backbone. At early epochs, G(z) produces spectrogram-like noise, while the supervised loss begins shaping the shared representation using the available real labeled examples. As adversarial training progresses and the generated samples become increasingly spectrogram-like, the backbone is exposed to a broader range of inputs through the critic branch, which can help regularize the learned representation. In turn, improvements in the shared representation benefit both the critic and the classifier. The interaction is therefore mediated by the shared feature extractor rather than by a direct classification loss applied to generated samples.

Without the generator, the critic branch would receive no synthetic input and the adversarial loss term would vanish. The architecture would then reduce to a supervised-only classifier trained through the BCE loss, which is functionally equivalent to the baseline CNN evaluated in Section 4.3. The comparison between both configurations therefore provides a direct measure of the generator’s contribution to the learned representation. A more controlled ablation, such as training the same dual-head architecture with the generator frozen, is planned for future work.

## 4. Experiments and Results

All models were implemented in PyTorch 2.0.1 + cu117 with CUDA acceleration on an NVIDIA Quadro RTX 4000 GPU. Audio processing relied on librosa [26] and SciPy [27], and classification metrics were computed with scikit-learn on the held-out test spectrograms.

### 4.1. SS-WGAN-GP Training Dynamics

**Figure 5:**
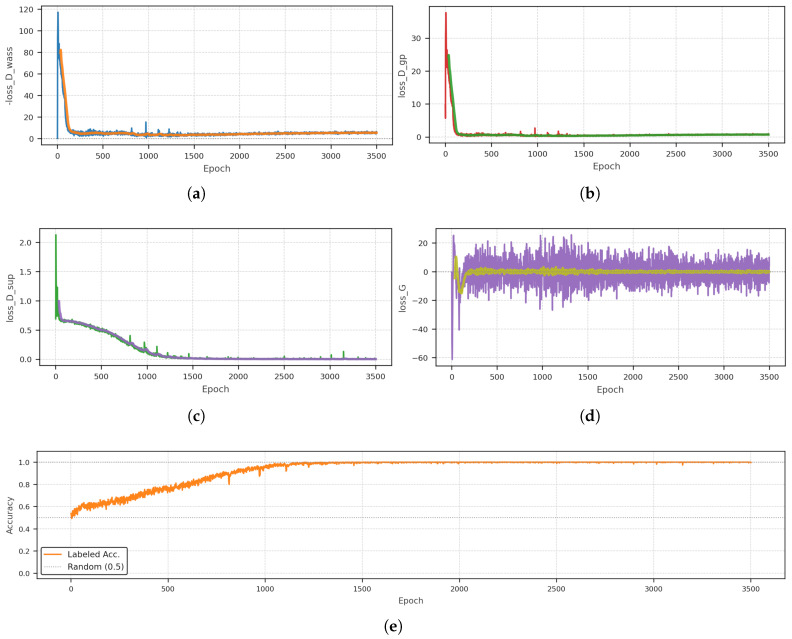
SS-WGAN-GP training history over 3500 epochs: (**a**) estimated Wasserstein distance, (**b**) gradient penalty, (**c**) supervised binary cross-entropy loss, (**d**) generator loss, and (**e**) classifier accuracy on the labeled real spectrograms. In panels (**a**,**b**,**d**,**e**), the lighter curve indicates a smoothed trend used to highlight the overall evolution of each metric.

The SS-WGAN-GP was trained for 3500 epochs. Figure 5 shows the complete training history. The estimated Wasserstein distance, gradient penalty, supervised loss, generator loss, and classifier accuracy were monitored throughout training to analyze the interaction between adversarial learning and supervised classification. The lighter curves shown in Figure 5 indicate the smoothed trend of each metric, facilitating visual interpretation of the overall training dynamics.

The estimated Wasserstein distance rises sharply during the initial training stage, reflecting the critic’s ability to distinguish real spectrograms from the generator’s initially poor outputs. After this transient phase, the curve stabilizes within a bounded range, suggesting that the generator progressively reduces the discrepancy with the real data distribution without causing unstable critic behavior. A similar pattern is observed for the gradient penalty: after an initial peak, it rapidly decreases and remains low throughout the rest of training, indicating that the Lipschitz constraint is enforced in a stable manner once the optimization enters its steady regime.

The supervised binary cross-entropy loss decreases monotonically and approaches zero as training progresses, showing that the classification branch learns to separate the labeled classes effectively. This behavior is mirrored by the classification accuracy on labeled data, which rises from near-random performance at the beginning of training to values close to 1.0 and then remains stable. Together, these two curves indicate that the shared representation learned by the discriminator becomes increasingly discriminative for the quality-classification task.

The generator loss exhibits large fluctuations during the earliest epochs, which is expected in adversarial training when the generator is still far from the target distribution. After this initial stage, it oscillates around a relatively stable range without diverging, which is consistent with a balanced adversarial interaction between generator and critic rather than with mode collapse or unstable optimization.

Overall, the training curves indicate that the proposed SS-WGAN-GP reaches a stable optimization regime in which the adversarial and supervised objectives can be optimized jointly. The critic remains well behaved, the supervised branch converges, and the generator continues to receive informative gradients throughout training. These observations support the feasibility of the proposed framework under limited-data conditions.

The reported model corresponds to the final training epoch (epoch 3500). Training was continued beyond the point at which the supervised loss stabilized in order to monitor the long-term stability of the adversarial dynamics and the visual quality of the generated spectrograms. Inspection of the loss curves confirmed that the Wasserstein estimate remained bounded, the gradient penalty stayed low, and the classification accuracy did not degrade in the later epochs. This procedure does not constitute a formal model-selection criterion; in future work with larger datasets, early stopping based on a held-out validation set with part-level stratification will be adopted.

### 4.2. Visual Quality of Generated Spectrograms

**Figure 6:**
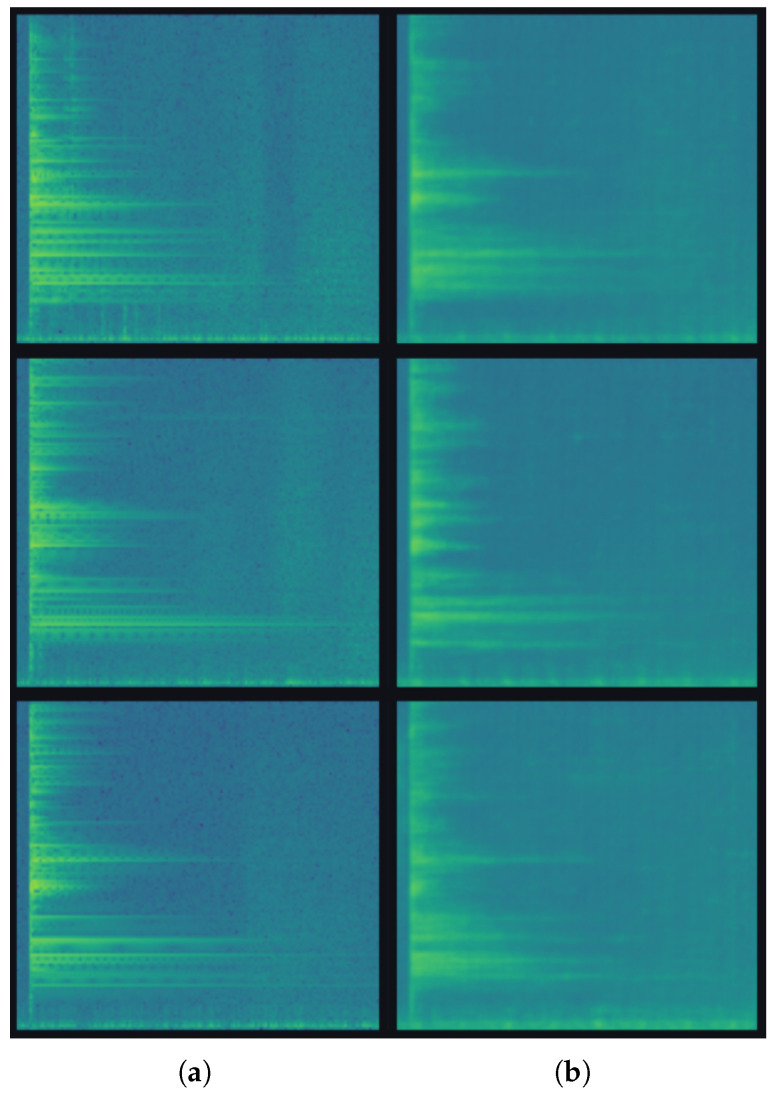
Representative real and generated log-spectrograms. The real samples (**a**) exhibit a strong initial energy concentration, progressive temporal decay, and a characteristic banded frequency structure. The generated samples (**b**) reproduce these same large-scale patterns, although with smoother local texture and less fine-grained detail. In both panels, the horizontal axis represents time after impact, the vertical axis represents frequency, and the color scale represents log-magnitude spectral energy, with darker colors indicating lower energy and yellow-green regions indicating higher energy.

Figure 6 presents representative real and generated spectrograms. A visual comparison shows that the samples produced by the trained generator reproduce the main macroscopic characteristics of the real acoustic responses. In both cases, most of the energy is concentrated at the beginning of the impact event and decays progressively over time, which is consistent with the temporal ring-down behavior expected in impact-response signals. The generated spectrograms also preserve the dominant banded structure visible in the real samples, indicating that the model has captured the principal frequency components associated with the resonant response of the part.

The generated images appear smoother than the real spectrograms. Fine-grained local variations and small intensity fluctuations are less pronounced in the synthetic samples, suggesting that the generator mainly learns the dominant global structure of the data distribution while attenuating higher-frequency detail and sample-specific irregularities. This behavior is consistent with the limited size of the training set and with the tendency of generative models in low-data regimes to prioritize stable large-scale patterns before reproducing subtle local texture.

Overall, the qualitative comparison indicates that the SS-WGAN-GP is able to generate spectrograms that are visually coherent and structurally consistent with the real acoustic data. The synthetic samples do not appear as arbitrary noise or collapsed repetitions, but rather as plausible members of the target distribution. This observation supports the idea that the adversarial branch exposes the shared backbone to additional spectrogram-like inputs during training, which may help regularize the learned representation used by the classification head.

While the visual comparison provides initial evidence of generation quality, it is not sufficient for rigorous industrial validation. Future work should quantify the similarity between real and generated spectrograms using feature-space distances computed on intermediate backbone representations, nearest-neighbor analysis to detect memorization of training samples, and controlled experiments comparing classifier performance with and without adversarial training. In the proposed framework, synthetic samples are not intended to replace real measurements or to be used as inspection evidence; their role is limited to the training phase, where they act as an implicit regularization mechanism for the shared feature extractor.

### 4.3. Classification Performance

The classification head $D_{c}$ of the trained discriminator was evaluated on the held-out real test spectrograms. The labeled dataset was split into training and test subsets using stratified sampling with a 75/25 ratio, yielding 88 training and 28 test spectrograms (15 conforming and 13 defective). This is the classifier produced directly by the proposed framework, without retraining, fine-tuning, or auxiliary models. For reference, a standalone lightweight CNN trained on the same real spectrograms without adversarial regularization was also evaluated on the same test set. The baseline CNN was trained for 100 epochs with Adam ($\mathrm{lr}=1\times 10^{-3}$, batch size 4) on the same training split used for the SS-WGAN-GP.

Table 2 reports the final performance of the SS-WGAN-GP classification head. On the held-out real test set, the proposed model achieved perfect classification performance, with an accuracy of 1.00 and precision, recall, and F1-score of 1.00 for both classes. All 28 test spectrograms (15 conforming and 13 defective) were classified correctly.

For comparison, Table 3 shows the results obtained by the supervised-only baseline CNN. The baseline reached an accuracy of 0.8929, with perfect precision for the conforming class but lower recall (0.80), indicating that some conforming samples were incorrectly predicted as defective. In turn, the defective class achieved perfect recall (1.00) but lower precision (0.81), reflecting the same tendency toward false-positive defective predictions.

The perfect accuracy reported in Table 2 should not be interpreted as an indication of flawless generalization, but rather as a consequence of the limited size of the test set, in which a single misclassification would shift accuracy by approximately 3.6 percentage points. Because the held-out test set contains only 28 spectrograms from a single production batch and defect population, the reported metrics have limited statistical strength. The more meaningful observation is not the absolute value of 1.00, but the consistent improvement of the SS-WGAN-GP classifier over the supervised-only baseline on the same data split, which suggests that the proposed semi-supervised strategy produces more robust decision boundaries under limited-data conditions.

The performance gap between the supervised-only CNN and the SS-WGAN-GP classifier can be attributed to the additional regularization imposed by the adversarial branch. While the baseline CNN optimizes only a supervised loss, the SS-WGAN-GP discriminator learns a shared representation that must support both real/fake discrimination and quality classification. This multi-objective training exposes the backbone to a broader set of spectrogram-like inputs through the generator and may lead to a more robust decision boundary under limited-data conditions. The supervised-only baseline could potentially be improved through conventional augmentation strategies such as time–frequency masking, additive noise injection, or mixup, as well as through architecture optimization or transfer learning from pre-trained audio models. These alternatives will be considered in future comparative studies.

Overall, the proposed SS-WGAN-GP outperformed the supervised-only baseline by 10.7 percentage points in accuracy on the held-out test set. Beyond the absolute numbers, the relative improvement over the baseline is the key observation: it suggests that the adversarially regularized semi-supervised training strategy helped the shared backbone learn more discriminative and better-balanced representations than supervised-only training under limited-data conditions. Importantly, this improvement was obtained while using the discriminator’s classification head directly as the final classifier, without any additional retraining stage. Although further validation on larger and more diverse datasets would still be desirable to confirm these findings, the present results provide supporting evidence that the proposed framework is a viable approach for acoustic quality inspection under extreme data scarcity.

## 5. Discussion

The observed training convergence of SS-WGAN-GP under extremely limited data conditions can be interpreted in light of two complementary factors. First, acoustic spectrograms of structurally similar parts exhibit strong regularity: energy distribution, temporal decay, and frequency-band patterns vary less across samples than in many natural-image domains, which reduces the effective complexity of the distribution to be learned. Second, the gradient-penalty formulation provides stable training signals even when the generated and real distributions remain far apart, a situation that naturally arises when only a very small number of real samples is available.

A standard GAN trained under the same data conditions would be expected to suffer from gradient saturation during early epochs, when the real and generated distributions are far apart. The Wasserstein formulation avoids this failure mode, which is particularly relevant when the training set is limited to a few tens of samples. An experimental comparison with alternative GAN formulations is left for future work.

A central idea of the proposed approach is that the generator does not need to produce perfect spectrograms in order to support the classifier. Even imperfect synthetic samples can act as a form of implicit data augmentation when they are processed through the shared backbone alongside real labeled images, potentially helping to regularize the learned representation and mitigate overfitting. In this sense, adversarial learning and supervised classification do not operate independently, but interact through the shared feature extractor used by both branches of the discriminator.

The final classification results support this interpretation. The classification head of the SS-WGAN-GP discriminator achieved perfect performance on the held-out real test set, whereas the supervised-only baseline CNN reached a lower accuracy. This difference suggests that adversarially regularized semi-supervised training helped the shared backbone learn a more discriminative and better-balanced representation than supervised-only training under the same limited-data conditions. Importantly, this performance was obtained using the discriminator’s classification head directly as the final classifier, without retraining, fine-tuning, or auxiliary models.

From an industrial perspective, the introduction of an acoustic quality-inspection stage before downstream remanufacturing operations can reduce unnecessary processing of non-recoverable parts and thereby lower both cost and environmental impact. At the same time, the present study still has important limitations. Although the test results are highly encouraging, the evaluation set remains relatively small, so broader validation on a larger and more diverse collection of parts would be desirable.

No minimum detectable defect size can be established from the available data, which does not contain a controlled series of defects with measured dimensions or severity levels. Determining detection thresholds as a function of defect geometry and severity would require a dedicated experimental campaign with artificially introduced or independently characterized defects. The model should be understood as a binary screening tool that reproduces the accept/reject decisions of the industrial quality-control process, not as a defect-sizing instrument.

Future work will extend the validation to larger datasets collected across multiple production batches, turbine housing variants, defect severities, and acquisition sessions to assess the statistical robustness and generalization capability of the proposed approach.

For practical deployment, the acquisition setup would need to be translated into a repeatable in-line or near-line inspection station with controlled impact energy, fixed microphone geometry, automated segmentation of impact events, and regular calibration. The main challenges include production-floor noise, variability in part positioning and fixturing, differences among TH variants and production batches, and domain shift between laboratory and industrial acquisition environments. A production-ready implementation would therefore require periodic revalidation and incremental dataset expansion using newly inspected and verified parts.

## 6. Conclusions

This paper presented an end-to-end pipeline for acoustic quality inspection of turbine housings under extreme data scarcity, based on a semi-supervised Wasserstein GAN whose discriminator also serves as the final quality classifier. The proposed SS-WGAN-GP combines the training stability of WGAN-GP with a supervised classification objective in a shared architecture designed for settings with very limited labeled data.

A central contribution of the work is the formulation of a training framework in which adversarial learning and supervised classification interact through a shared backbone representation. Within this framework, generator-produced spectrograms provide an additional source of spectrogram-like inputs during training, while the supervised branch encourages the learned representation to remain relevant for quality discrimination. This design makes it possible to train a classifier directly from the discriminator without requiring external unlabeled data, manual labeling of synthetic samples, or post hoc retraining.

Experiments on real TH impact-response data showed that the proposed approach generates visually coherent spectrograms, converges stably during training, and achieves strong classification performance on held-out real samples. In particular, the classification head of the trained discriminator outperformed the supervised-only baseline CNN on the test set, supporting the value of adversarially regularized semi-supervised learning for acoustic quality inspection under industrial low-data conditions.

Overall, the results indicate that SS-WGAN-GP is a promising approach for acoustic industrial inspection when labeled defective data are scarce. Future work should extend the evaluation to larger and more diverse datasets and investigate additional quantitative criteria for assessing generation quality.

## Figures and Tables

**Table 1:** Dataset composition from real audio recordings.

Category	Parts	Impacts/Part	Spectrograms	Spectrograms After Cleaning
Conforming	14	5	70	63
Defective	13	5	65	53
Total	27	—	135	116

**Table 2:** Classification performance of the SS-WGAN-GP discriminator on the held-out real test spectrograms.

Class	Precision	Recall	F1	Support
Conforming	1.00	1.00	1.00	15
Defective	1.00	1.00	1.00	13
Accuracy	1.00			28

**Table 3:** Performance of the baseline lightweight CNN trained on real spectrograms only and evaluated on the same held-out test set.

Class	Precision	Recall	F1	Support
Conforming	1.00	0.80	0.89	15
Defective	0.81	1.00	0.90	13
Accuracy	0.8929			28

## Data Availability

The datasets presented in this article are not readily available because they contain proprietary industrial data from an internal company production process and are subject to confidentiality restrictions. Requests to access the datasets should be directed to the corresponding author.
